# Reconfiguring health workforce: a case-based comparative study explaining the increasingly diverse professional roles in Europe

**DOI:** 10.1186/s12913-016-1898-0

**Published:** 2016-11-08

**Authors:** Antoinette de Bont, Job van Exel, Silvia Coretti, Zeynep Güldem Ökem, Maarten Janssen, Kristin Lofthus Hope, Tomasz Ludwicki, Britta Zander, Marie Zvonickova, Christine Bond, Iris Wallenburg, On behalf of the MUNROS Team

**Affiliations:** 1 Institute of Health Policy and Management, Erasmus University Rotterdam, Rotterdam, Netherlands; 2 Postgraduate School of Health Economics and management (ALTEMS), Universita Cattolica del Sacro Cuore School of Economics, Milan, Italy; 3 Faculty of Economics and Administrative Sciences, TOBB University of Economics and Technology, Ankara, Turkey; 4 Uni Research Rokkan Centre, Bergen, Norway; 5 Faculty of Management, the University of Warsaw, Warsaw, Poland; 6 Faculty of Economics and Management, Technische Universität Berlin, Berlin, Germany; 7 Third Faculty of Medicine, Charles University, Prague, Czech Republic; 8 Division of Applied Health Sciences, University of Aberdeen, Aberdeen, Scotland

**Keywords:** Skill mix, Health workforce, Europe, Extended roles, Advanced roles, Comparative study, Health care teams

## Abstract

**Background:**

Over the past decade the healthcare workforce has diversified in several directions with formalised roles for health care assistants, specialised roles for nurses and technicians, advanced roles for physician associates and nurse practitioners and new professions for new services, such as case managers. Hence the composition of health care teams has become increasingly diverse. The exact extent of this diversity is unknown across the different countries of Europe, as are the drivers of this change.

The research questions guiding this study were: What extended professional roles are emerging on health care teams? How are extended professional roles created? What main drivers explain the observed differences, if any, in extended roles in and between countries?

**Methods:**

We performed a case-based comparison of the extended roles in care pathways for breast cancer, heart disease and type 2 diabetes. We conducted 16 case studies in eight European countries, including in total 160 interviews with physicians, nurses and other health care professionals in new roles and 600+ hours of observation in health care clinics.

**Results:**

The results show a relatively diverse composition of roles in the three care pathways. We identified specialised roles for physicians, extended roles for nurses and technicians, and independent roles for advanced nurse practitioners and physician associates. The development of extended roles depends upon the willingness of physicians to delegate tasks, developments in medical technology and service (re)design. Academic training and setting a formal scope of practice for new roles have less impact upon the development of new roles. While specialised roles focus particularly on a well-specified technical or clinical domain, the generic roles concentrate on organising and integrating care and cure.

**Conclusion:**

There are considerable differences in the number and kind of extended roles between both countries and care pathways. The main drivers for new roles reside in the technological development of medical treatment and the need for more generic competencies. Extended roles develop in two directions: 1) specialised roles and 2) generic roles.

**Electronic supplementary material:**

The online version of this article (doi:10.1186/s12913-016-1898-0) contains supplementary material, which is available to authorized users.

## Background

In response to both shortages in workforce and the persistent rise in spending on health care and its workforce, national authorities and health care organisations have endorsed inter-professional work and task substitution [1, 2]. Consequently, healthcare workforce has diversified in several directions with formalised roles for health care assistants, specialised roles for nurses and technicians, advanced roles for physician associates and nurse practitioners, and new roles for new services, such as case managers [3, 4]. This workforce reconfiguration exemplifies a growing international trend in healthcare policy to redistribute resources on the basis of professional accomplishment rather than historical workforce hierarchies and roles [3, 5].

In the literature on workforce reconfiguration and new roles development, professional jurisdiction is key [2, 6]. This concept was coined by Andrew Abbott in the late 1980s, to indicate professions’ right to control particular services and activities [7]. Professional groups can claim exclusive authority because their work is grounded in exclusive knowledge, including the indeterminate and experiential knowledge that is tacit in nature, yet situated and embodied in practice [8–10]. Abbott analysed how professionals defend themselves and respond to competing claims from neighbouring professions. He points out that professions constantly engage in jurisdictional disputes, producing occupational vacancies. Furthermore, jurisdictional claims are mediated by negotiations at the public, the legal and the workplace level. While jurisdictional claims are rigid at the public or legal level, they may be more fluid in the workplace where daily work needs to be done. Here professional boundaries cannot be strictly maintained, which impacts on the profession’s wider jurisdictional claims [2, 7, 11]. Following Abbott, the analysis of the tasks or workforce activities is crucial to understanding changes in professionalization.

A growing body of literature shows the impact of the introduction of new roles on jurisdictional claims [1, 2, 6, 12]. Most of this work offers insight into the strategies deployed by vested professions protecting their jurisdictional boundaries, for instance by discarding less pleasant and stigmatising work to others while retaining the more desirable work [13]. Others, conversely, have shown that professions seek to maintain organizational work to enhance authority over clients, demonstrating how the more mundane and considerably non-expert activities actually generate important resources for exerting authority and protecting autonomy [14]. Moreover, Martin and colleagues argue that advances in new technologies and managerial practices alter the character of professions [5]. Technological developments contribute to the explication of knowledge, threatening the claim that (expert) knowledge is irreducible and transcending in its constituent parts.

Managerial reforms, in turn, have introduced new connections between professionals and (bureaucratic) organisations [15–17], rendering organisations a vehicle for professional action and professional development [15, 18]. This increased connectivity or ‘intertwinement’ of professions and organisations is described in the literature on ‘organised professionalism’ [15, 17, 19–21]. This literature portrays organisations as key sites for professional development and regulation. Accounts of change point towards levels of increased bureaucracy and formalisation, leading to new practices of standardisation and accountability [22]. Professional work is increasingly shaped by the interests and routines of employing organisations – aligning professional expertise with organisational and commercial needs [23]. Recently, Noordegraaf has argued that professions not only respond and adapt to an organisation’s needs, but also incorporate the organising work themselves, redefining ‘organising’ as one of their core competences [24].

How does this more dynamic evolvement of professional roles, leading to more diversity in professional work and hence shifting professional jurisdictions, play out in practice? The exact extent of this diversity across Europe is yet unknown, as are the drivers of this change and their interactions. This paper provides insight into the evolution of extended professional roles in Europe and examines the drivers of extended professional roles. We use ‘extended professional roles’ to indicate both the development of new roles for existing professions and the introduction of new professions into the health service. In this paper, we compare the diverse roles in the care pathways of breast cancer, heart disease (post STEMI) and type 2 diabetes, and drivers of the skill mix in healthcare in eight European countries. The research makes part of an extensive and ongoing research project, MUNROS, on the impact on practice, outcomes and costs of new roles for health professionals.^1^

This paper aims to clarify the development of new professional roles in Europe, both its scope and orientation. In doing so, we follow a two-fold analytical approach. First, we map the emerging extended professional roles in Europe. Second, we provide insight into the social processes that drive the development of these extended roles. The three questions guiding the research are:

1. What extended professional roles are emerging in health care teams?
2. How are extended professional roles created?
3. What are the main drivers that explain the observed differences, if any, in the skill mix in and between countries?

## Methods

This paper draws on 16 ethnographic studies in eight European countries (Czech Republic, Germany, Italy, Poland, Netherlands, Norway, Scotland and Turkey), in which we studied extended professional roles development for three clinical conditions: breast cancer, heart disease (post ST-segment elevation myocardial infarction (STEMI)) and type 2 diabetes. Below, we explain the case-based comparative research design, the data collection and the data analysis.

### Case-based comparison

We conducted case studies in a purposive sample of eight European countries based on maximal case variation [25]. Sampling took place prior to the start of the MUNROS study [26]. The selected countries represent the different stages in the reform of health care delivery systems. We distinguished three groups of countries: 1) countries at the forefront of innovative delivery systems; 2) countries with established and stable delivery systems; 3) countries at the back front of innovation in delivery systems (see Table 1). The healthcare systems in the eight countries include both tax-financed and social insurance-financed systems, as well as centrally organised and regionally-based systems – with and without competition among insurers.

**Table 1:** Selection of countries

Countries at the forefront of innovative delivery systems	Countries with stabilized delivery systems	Countries at the backfront of innovative delivery systems
England, the Netherlands, Scotland	Germany, Italy, Norway	Czech Republic, Poland, Turkey

The reasoning behind maximal case variation was to encapsulate 1) countries with new professions bearing the legal power and authority to establish a new working domain, i.e., the Netherlands and Scotland; 2) countries with new professional roles, i.e., the Czech Republic, Germany, Italy; and 3) countries where new professions and professional roles appear to have a marginal role, i.e., Norway, Poland and Turkey.

To ensure comparability we focused on three conditions and their associated care pathways, allowing us to order the data and structure the differences and similarities. A pathway includes all care within a hospital organisation, carried out by any healthcare professional, provided to a patient from diagnosis through to long-term follow-up.

### Selection of care pathways

We used information-oriented sampling to select the care pathways, aiming to pick those pathways where care is delivered by various professionals, including either a new profession (e.g., a physician associate or a nurse practitioner) or existing professions in extended roles (e.g., a specialist nurse or a technician) in at least one of the participating countries. In addition, we aimed for care pathways that had a European-wide consensus on the clinical treatment [27] and the selected diseases had to be a high burden to society. Finally, to be able to measure the impact of extended roles in later stages of the MUNROS project, we aimed for care pathways which routinely had data available on outcomes, i.e., process outcomes, intermediate outcomes and patient outcomes. For an overview of the selection criteria see Table 2.

**Table 2:** Selection criteria

Criterion	Operationalisation
Care could be delivered by a range of health professionals.	• In at least some of the partner countries, care is delivered either by new professions or in new roles for existing professions.
	• The contribution of various professions differs across partners.
Burden to society.	• High prevalence of clinical condition.
	• Significant morbidity and mortality are associated with the condition.
Routine data available on health outcomes.	• Outcomes of processes.
	• Intermediate outcomes.
	• Clinical outcomes.
Except for new professional roles, procedures and clinical management are similar across national boundaries.	• Care is delivered in primary and secondary settings.
One selected care pathway will mainly be located in the primary care setting.	• Other selected care pathways will mainly be in the secondary care setting.
It is desirable that one care pathway involves:	• Acute and chronic management and surgical procedure.
	• Prevalence among those aged 65 and over.
If possible one pathway might involve:	• Predominantly a need for generalist skills.
	• Predominantly a need for specialised skills.

To facilitate the final selection we organised a two-day invitational workshop with experts from various medical and management fields to discuss a list of seven potential care pathways identified by the MUNROS partner countries, in conjunction with their local Country Expert Advisory Groups, as well as an international Expert Advisory Board. Based on the criteria in Table 2, three care pathways were selected: breast cancer, heart disease (post STEMI) and type 2 diabetes. The three clinical conditions are examples of: a condition requiring a scheduled surgical intervention followed by post-operative care and monitoring; an emergency requiring unscheduled hospital care, rehabilitation and long term care; and a chronic condition managed largely in primary care. Care pathways were distributed such that all participating countries covered two care pathways and each pathway was covered by at least half of the countries, taking into account the occurrence of extended roles in each country (i.e., the sampling criteria) (Table 3).

**Table 3:** Selection of care pathways

Country	AMI	Breast cancer	Diabetes II
Czech Republic	x	x	-
Germany	x	x	-
Italy	x	-	x
The Netherlands	x	x	-
Norway	x	x	-
Poland	x	-	x
Scotland	-	x	x
Turkey	-	x	x
Total	6	6	4

### Data collection

Ethnographic research was conducted by a team of 11 researchers in all countries on the selected pathways, resulting in a total of 16 case studies (Additional file 1). The data was collected between February and August 2014. The ethnographic approach provides in-depth insight into the introduction and development of extended professional roles in day-to-day clinical practice. We sought to include a wide variety of extended professional roles. Hospitals were identified and selected according to rurality, specialisation and size. Rural hospitals, less specialised and smaller hospitals are more likely to employ staff who practice across a wider range of conditions and use a wider range of competences. In contrast, large teaching hospitals with specialised services are more likely to employ specialised professional roles (e.g., specialised nurses) and specialised technicians.

Data collection included in-depth semi-structured interviews and observations (Additional files 2, 3, 4 and 5). We interviewed physicians to understand which tasks they would be willing to delegate and under what conditions. We interviewed nurses and other care professionals- such as care assistants and technicians but also physician associates and advanced nurse practitioners-to understand if and how their roles have been extended, how extended roles are created and how different care professionals interact, investigating the jurisdictional aspects of role development. In addition, we interviewed team managers and human resource managers to understand why and how extended roles are deployed in an organisation. Finally, we interviewed patients to find out how they perceived the care provided by extended roles. In total, 160 interviews were conducted. The duration between the interviews varied between 20 min up to 90 min. With the permission of the interviewees, we recorded and transcribed the interviews verbatim.

The research team observed healthcare professionals in their daily work (see Table 4). Through observing care professionals in various roles we developed an empirical understanding of the sociocultural contexts, processes and sense-making activities significant to these professionals [25, 28]. During the observations, field notes were made that were typed up in detail immediately after the observation period, leading to ‘dense descriptions’ of daily work practices [29, 30].

**Table 4:** Number of interviews and observations

Country	Number of interviews	Number of observations	
		Days	Hours
Czech Republic	34	24	62
Germany	14	7	35
Italy	19	30	261
Norway	23	11	47
Poland	12	7	41
Scotland	10	6	25
The Netherlands	27	18	116
Turkey	21	9	30
Total	160	112	617

Prior to the study, we defined a case study protocol, targeting the number of respondents and defining both a topic list for the interviews and an observation protocol. All country teams interviewed and observed the minimum number of respondents. Hence, all data were included in the analysis. The research teams from the Czech Republic, Italy and the Netherlands conducted more interviews and observations, which enabled to enrich the data set.

### Data analysis

Data analysis occurred in two steps, leading from a structured, country-specific analysis to a cross-site analysis. To ensure comparability between sites and between countries, a consistent data analysis approach was developed. During the data collection and analysis, researchers discussed issues emerging from data collection and intermediate results at monthly online meetings and during two face-to-face workshops [31, 32]. At the first workshop, researchers were updated on the approaches to take in interviewing and observation. At the second workshop, researchers presented a selection of observations and interview quotes to gain in-depth understanding of the various case studies. Using the literature and case study results, a list of codes was developed and agreed on as the final common coding framework. Partners attending this workshop were asked to come to shared insights into the everyday work of extended roles and the distribution of tasks and responsibilities. All partners presented at the workshop in a standardized format:

1. The role of new professionals in the care pathways.
2. The scope of practice of the new professional roles.
3. A description of the work of new professional roles in practice.
4. A description how tasks were distributed between the professionals in the care pathway.

The two workshops and monthly Skype meetings ensured both comparability and sensitivity for local differences. Moreover, it enabled the two research coordinators (the first and last author, respectively AdB and IW) to come to grips with the differences and similarities in extended roles development in the different countries. Subsequently, each research team in each country prepared their own country-level report. In the country report the partners described: the policy context, the payment system, the legal system, the scope of practices of new roles, the hospital at which the study was conducted, the sequence of tasks in the care pathways, a flow chart of the care pathway, the process of task redistribution, the mundane work to coordinate care and tasks within the care pathways. All sections of the case report included ample and extensive quotes and excerpts from the field notes.

The current paper is based on a cross site analysis using the country reports. AdB and IW compared the different sections of the case reports to map the differences between the countries and between the care pathways. We coded the quotes and field excerpts that revealed 1) how the different professionals – physicians, nurses and new professional roles – claimed authority over tasks, 2) how the distribution of tasks became fluid to get things done; 3) how new professional roles created room for new tasks; 4) how physicians responded to claims new professional roles made over tasks; 5) the standardization of knowledge and its implication for task distribution; 6) the standardization of tasks and its implication for task distribution; 7) the distribution of organising work.

We presented the preliminary findings of the coding to the whole MUNROS research team and the external advisory team. The results of the analysis were shared and discussed with sociological scholars, policy makers and the professional bodies for physicians, nurses and new professional roles.

## Results

We present our findings under three headings, corresponding to the three research questions. Under “What are the emerging extended roles of healthcare professionals in healthcare teams?” we categorise the extended roles that have been introduced in the participating countries. Under “How are extended roles created?” we describe how the extended roles were created in healthcare practice, revealing the fluidity of new professional role development. Finally, under “What are the drivers that explain differences in skill-mix?” we describe the main drivers of extended professional roles and explain the main differences in skill mix. We illustrate the results with quotes and extracts of observational field notes from the case studies.

### What are the extended roles of health care professionals?

This section gives an overview of the extended roles of health care professionals in the three care pathways (see Table 5). We observed considerable differences in both the number and the kind of roles in the three care pathways. In the heart disease care pathway we identified 20+ roles in the eight countries. We identified new roles for physicians –both more specialised roles and more general roles. An example of a specialised role is the intervention cardiologist. An example of a general role is a hospital physician or an outpatient clinician. The diversification of physicians mirrors the diversification of nurses; with a CCU [coronary care unit] physician comes for example a CCU nurse. In addition, we identified several technicians, such as a paramedic, a radiographer, a resuscitator or a perfusionist, and several assistants.

**Table 5:** Various roles in the three care pathways

Stage	Role	AMI	Breast cancer	Type 2 Diabetes
Diagnosis	Advanced nurse practitioner		x	
	Breast clinician	x	x	
	Cath Lab nurse	x		
	Diabetologist			x
	Emergency cardiologist	x		
	Emergency nurse	x		
	Emergency physician	x		
	Nephrologist			x
	Paramedic	x		
	Pathologist		x	
	Physician associate	x		
	Radiographer		x	
	Resuscitator	x		
	Technician	x		
	Triage nurse	x		
Treatment	Advanced nurse practitioner		x	
	Cath Lab nurse	x		
	CCU cardiologist	x		
	CCU nurse	x		
	Documentation assistant	x		
	Educationalist			x
	Health care assistant	x	x	
	Hospital physician	x	x	
	Intensive care nurse	x		
	Intervention cardiologist	x		
	Perfusionist	x		
	Radiologist		x	
	Radiology physicist		x	
	Radiology technician	x		
	Technical radiation assistant	x	x	
	Ward nurse		x	
Follow-up	Advanced nurse practitioner		x	
	Breast care nurse		x	
	Diabetes education nurse			X
	Dietician			X
	Documentation assistant	x		
	Foot care technician			X
	Health care assistant	x		
	Hemodynamics nurse	x		
	Intensive care physician	x		
	Nutrition specialist	x		X
	Ophthalmologist			X
	Consultant Palliative care		x	
	Out-patient clinician	x		
	Out-patient nurse	x	x	
	Podiatrist			X
	Retinal screening nurse			X

**Table 6:** Classification of care pathways

Country	AMI			Breast cancer			Type 2 Diabetes		
	No new roles	Specialised roles	Organising roles	No new roles	Specialised roles	Organising roles	No new roles	Specialised roles	Organising roles
Czech Republic	-	X	-	-	x	-	-	-	-
Germany	-	-	x	-	x	x	-	-	-
Italy	-	-	x	-	-	-	-	x	x
Norway	x	-	-	x	-	-	-	-	-
Poland	-	X	-	-	-	-	-	x	-
Scotland	-	-	-	-	x	-	-		x
The Netherlands		X		-	x	x	-	-	-
Turkey	-	-	-	-	x	-	-	x	-
Total	1	3	2	2	4	3	1	2	2

In contrast to the heart disease care pathway, the type II diabetes care pathway has much less diverse roles. Here, we identified 11 roles. In this care pathway, we identified for example an educationalist, a podiatrist and a food care technician. In the breast cancer care pathway we identified fewer roles than in the heart disease care pathway, but more than in the type II diabetes care pathway. Here, we identified several nursing roles – such as advanced nurse practitioner, oncology nurse, ward nurse, breast care nurse and an out-patient nurse.

In all countries we identified new roles (see Table 6). In countries at the forefront of innovative delivery systems –both Scotland and the Netherlands, we identified advanced roles such as the physicians associate and the nurse practitioner. Interestingly, in countries at the back front of innovation in delivery systems we identified as many new roles as in the other countries. Especially, in Czech Republic and Poland we identified mostly specialized roles for physicians, nurses and technicians. In countries with established and stable delivery systems (Germany, Italy), we identified both specialised roles en organising roles – such as documentation assistant, case manager and hospital physician.

A cross country comparison reveals the diversity in tasks among professionals with the same job title, e.g., specialist nurses, paramedics, and radiation technicians. Hence, the exact role of a professional, including tasks, competencies and responsibilities may differ between countries. This implies that although the findings from this study provide insight into the increasing diversity in individual roles in three distinct care pathways, they cannot easily be generalised to other care pathways.

Furthermore, the diversity in roles complicate the comparison of extended roles across countries. We cannot compare the roles in a care pathway based upon job titles. Yet, and following Abbott’s [7] insight of the importance of studying actual tasks and work activities to the understanding of professional role development, we need to make detailed comparison of these tasks and activities. We compare the diversity of health care delivery systems in which new roles develop. Hence, studying the development of new roles in diverse contexts is the main advantage of this large international study on new roles.

### How are extended professional roles created?

Following this overview of the various roles in the three care pathways in the eight countries, we now turn to the question how extended roles evolve. From the interviews it was apparent that physicians play a key role in the distribution of work. Extended professional roles depend on local and sometimes even individual professional arrangements – despite protocols or, in some cases, a legally defined scope of practice. In the Dutch heart disease case study a physician associate – a master’s-level practitioner operating in the medical domain and possessing the legal right to conduct clinical procedures that used to be the preserve of medical doctors, e.g., endoscopy, defillibration and prescribing medicine [33] – explained this dependency as follows:

*“It is up to the cardiologist who implants the device to decide how much I can do during an operation. Cardiologists differ in what they leave up to me (…)”* (physician associate, cardiology, Netherlands)

So, tasks and responsibilities depend strongly on the personal relationship between the health care professional aspiring to take on an extended role and an individual physician. Trust in the competences of an *individual* practitioner plays a crucial role herein. For example, in the Czech case study of heart disease nurses distinguished “their” doctor (an interventionist cardiologist) from the physicians of the CCU department:

“*When I have to call the nurse from CCU, she assists her doctor and I assist mine*” (nurse, catheterization laboratory (Cath Lab), Czech Republic cardiology case)

*“Sometimes we feel as in MASH. I often think of a doctor from another place who was helping us. At night, when we were admitting a patient with AMI, he came and asked me to call in the team. I had to tell him that he and I are the team. He was quite surprised and later on valued my critical thinking when I, in certain phase of the procedure, had prepared the defibrillator. He offered me a job on the clinic he worked at but I had to decline it. We are trained that way, we can do it, we can organize the work very well*”(nurse, Cath Lab, Czech Republic cardiology case).

Personal relationships often build up over years. Several professionals in extended roles, such as specialised nurse or advanced nurse practitioner, already had extensive experience as a nurse or technician. The physicians knew them well before they started training and were entrusted with the new role, which also meant a new career opportunity:

“*I started here in’95. I’d already been working here for ten years when someone asked me if I wanted to do more in my scope of practice. They offered me a position as PA.*” (physician associate cardiology, Netherlands)

The kind of work and clinical responsibilities delegated to professionals in extended roles differ not only among individual practitioners, but is also situated. The case studies reveal that often the need ‘to keep things going’ [11] encourages the introduction of new roles. The next excerpt from the Polish heart disease case illustrates how an extended role moves beyond its initially defined scope. Here, paramedics, educated as technicians, work in the hemodynamics department. Although not officially on the emergency team, they can join it temporarily:

*“If anything goes wrong I run into the room. So I’m involved in saving lives if necessary. When the [the heart] stops I run to reanimate the patient. And we all [cardiologist, nurse, technician/paramedic] act together. (…) Defibrillation or intubation? I can do that!”* (technician/paramedic, Poland)

Extended professional roles may in reality cover a wider range of expertise and related activities than formally described. The fluidity of the workplace [7, 11] enables individuals to participate in complex or clinically unstable situations. Yet what they actually *do* also depends on their professional background and acquired competences:

*“Now we’ve got physician associates who come from all sorts of backgrounds. They may go and see somebody first, come back and then get the surgeons to come in. You know, after they’ve done kind of all the ground work. On the wards, they may take blood. They have a bigger, broader remit and, really, their roles are quite flexible. It’s really what the service needs. In places they’d act as an assistant in surgery. They are roles in their own right but they go across quite a wide range, from wards to theatres to clinics.…” (manager, Scotland).*

Besides personal relationships, also formal relationships play a role. In Italy, nurse assistants played an increasingly important role. However, as the assistants were employed by an external company, nurses were very careful handing over tasks:

*“Even if Luisa has the competences for administering the oral therapy to patients, always under the supervision of nurses, she is not allowed to do this on this ward.[…] I think it is because their role are not properly defined yet, and nurses usually do not trust them.” (Observation – Hospital Care, Italy)*

Hence, locality or ‘situatedness’ plays a crucial role in the (re)allocation of tasks. This sometimes results in practitioners not practicing to their full (legal) competence. The case studies reveal that legally assigned clinical activities sometimes cannot be carried out due to restrictive local arrangements. In countries where extended professional roles are legally recognised – such as the advanced nurse practitioner in the Netherlands and Scotland, these new professions find it hard to make full use of their new authority in actual care provision, like medicine prescription. Interviewees revealed that administrators, physicians, nurse practitioners and physician associates were often not aware of national (legal) regulations that provided nurse practitioners with more (legal) space to practice independently from physicians, or they accepted the organisation’s or physician’s wish to not (fully) use their legal space to act as they did not want to harm the relationship of trust that allowed them to work independently in other parts of the care pathway.

The differences in tasks and responsibilities, the organisational embeddedness of practitioners, and the situatedness of the work, limit further development of extended professional roles, and may even lock professionals into their work place. Furthermore, extended roles that cannot be linked to a body of expert knowledge or that lack a national infrastructure, hinder moving between care pathways and organisations.

### What main drivers explain the main differences in the skill mix?

This section describes two directions in which extended roles develop that emerged from our analysis: specialisation which involves an increasing level of expertise in a narrowly defined area, and generalisation which concentrates on organising activities.

Specialisation enables professionals to extend their role and to carry out clinical tasks relatively independently from physicians as they develop expertise and clinical routines in a particular clinical area. Examples are: designing a radiation therapy plan, stabilising patients in emergency care centre and monitoring type 2 diabetes patients. An example of a specialised technical role is the hemodynamic technician. In the heart diseases (post STEMI), we interviewed a Polish hemodynamic technician who followed a formal training and is therefore allowed to perform a specialised technical task. In the following quote he contrasts his former role as a paramedic with his new specialised role in hemodynamics:

*“There is a requirement that the equipment has to be serviced by a technician. So, we all have the required training and are certified. We all had radiology training and got a certificate that is valid for five years. At the same time we are all paramedics. Actually, I first worked as a paramedic and only afterwards started to work in hemodynamics section”* (Technician, Poland)

In all three care pathways, we observed specialised physicians who worked with specialised nurses and specialised technicians. Physicians, we expected, protect their jurisdiction by distributing non specialised work to others. Yet, nurses and technicians specialise too. Hence, the care pathway can be depicted as a sequences of relative small but specialised tasks. This is well illustrated in a field note excerpt about the work of Czech radiation assistants (RA) who position the patient during radiation treatment. The development of a treatment plan – as the field note below shows, transcended in parts, such as perform a CT scan with a fixed number of cuts, insert CT scan into planning system, create 3D reconstruction of the body, draw bone structure, etc. Moreover, the treatment is designed as a managerial process. The physician defines the treatment plan which the RA develops into a radiation plan. After the physician approves the radiation plan, the RA can start the simulation to fill in the planning system:

*One important condition for correct radiation treatment is the positioning (fixation) of the patient to ensure a precise, stable and easily reproducible position. This is done by an RA. The RA performs a CT scan, including a series of transverse CT cuts that provide information about the placement of the tumour deposit and density of tissues. This occurs under the supervision of a physician. The number of CT cuts is defined by the physician. After the CT screening, the RA imports the CT images into the planning system, creates a 3D reconstruction of the patient’s body and draws the outline of the body and bone structures. Next, the physician defines the treatment plan, setting the patient’s position and applied fixation tools, the target dose, and dose limits for critical organs. On the basis of these data the radiology physicist or the RA produces the radiation plan. When the physician has approved the radiation plan, he sends the plan for simulation to the RA, who checks the radiation fields and the position of lamellas at the multilamellar collimator. After the simulation takes place, the parameters of individual fields and data from the planning system are sent to the irradiator. This enables automatic setting of the specified parameters (table position, shoulder slope, field size, number of monitor units,* etc.*) for the given patient.* (Hospital field notes, Czech Republic)

As nurses and technicians specialise too, they also delegate tasks to others. This is nicely illustrated by the Scottish case where podiatrists and foot therapists provide care to diabetic patients suffering foot injuries. The physicians delegate foot care to the podiatrist, who in turn further delegates the screening aspect of foot care to foot therapists. Podiatrists cover a wider part of the care pathway; they run a diabetic foot service, take care of high-risk patients, order diagnostic tests, prescribe drugs, and conduct wound care. They delegate part of their work to the foot therapist who annually screens low-risk patients for foot complications arising from diabetes.

Generalisation involves both the organisation of care and the integration of nursing care and medical care. Professionals in organising roles work independently of physicians as they run the services on their own. Hence, rather than resulting from task delegation, the extended roles developed when hospitals started to offer a new health service. In other words, service redesign enables professionals to extend their role, to carry out tasks independently of physicians, and to contribute to the efficiency of care. These health services serve both organisational (i.e. increasing capacity and productivity) and medical purposes (lower admission rates, shift to out-patient care, and focus on lifestyle to improve patient health outcomes). Examples are: the cardiac rehabilitation nurse practitioner, the case manager, the triage nurse, the breast clinician and the out-patient physician. These new roles cover a more extensive part of the pathway. In Turkey, for instance, oncology nurses have an important role in guiding and supporting the patient through the breast cancer care pathway. They organise the care and support the patient. Davina Allen suggests this ‘organising work’ is ‘nursing work’ [34]. More than ‘just organising’ as a more or less rational activity, nurses ‘translate’ patient needs as they modify and adjust needs and requirements to secure a match between the needs of a patient and the available services [35]. This not only indicates a broader view of the definition of care provision in the care pathway, it also reveals how nurses in their extended role mediate and integrate ‘cure’ and ‘care’.

Some new professionals - such as advanced nurse practitioners, physician associates and specialised nurses - incorporate a generic perspective into their work, considering and responding to the wider organisational aspects of patient treatment. An example is the advanced nurse practitioner in the Netherlands. Advanced nurse practitioners combine caring tasks with clinical activities previously carried out by physicians. The next excerpt shows how the nurse practitioner provides clear instructions to the patient on drug treatment, mediating optimal clinical care and the patient’s well-being. This advanced nurse practitioner includes the well-being of the patient as a part of the medical treatment. She balances the risk of infectious diseases against the side effects of medicine that boosts the immune system:

*The next patient enters the consulting room with her husband. The nurse practitioner (NP) immediately notices her pale skin. ‘We need to check your blood sample!’ The woman says she feels terrible. She had an awful headache last time, and has been crying for the past 24 h. She can’t take it anymore. The NP nods sympathetically: ‘I think it was the Neulasta’ [a drug that can be administered during chemotherapy to strengthen the immune system]. She suggests stopping the drug: ‘It’s your last course today.’ The patient asks about the risk of not taking it. The NP answers that there is only a small risk of infection. The patient has had a throat infection before and ‘the GP wouldn’t give me anything for the pain.’ The NP says that she shouldn’t call the GP: ‘You need to call us! Anytime! Do you have the number? Or ask your GP to contact the hospital. There’s always someone around.’ The patient hesitates: ‘What should I do? I think I’ll just take it.’ The NP advises her not to take it: ‘You’ll be very sick again. It’s your last course’.* (Hospital field notes, the Netherlands)

While new technologies facilitate the development of specialised roles, the development of new generic or ‘organising professional roles’ is linked to an increasing demand for care as well as the introduction of new treatment facilities. In the Dutch heart disease case, for instance, the number of patients treated for STEMI increased from 2,000 to 9,000 per year. Hospital admission time was greatly reduced. Whereas patients used to stay in the hospital for three days, they are now discharged after only a few hours of treatment. Both the increased volume and the enhanced complexity of care have implications for workforce capacity and work content in terms of expertise and organisation of treatment. Patients discharged early need more complex care at home. For example, some discharged patients continue to receive intravenous therapy in the domicile. In the Dutch case study, most cardiologists only saw the new patients, and delegated out-patient care to nurse practitioners and physician assistants:

*“Nurse practitioners are the contact person for patients on the heart rehabilitation trajectory. I totally support that. Those patients are often very unsure about their disease and often have lots of questions. It takes a lot of time before you can discuss them during an appointment with a cardiologist. It’s so nice that these new professionals are available to answer these kinds of questions. It helps that these professionals see those patients every now and then. So the care provider is always the same person (and) their biggest strength is that their care is very accessible and approachable.”* (cardiologist, the Netherlands)

In this case, nurse practitioners play an important role in supporting patients during extended care at home. Patients can reach them easily with questions or worries, something we frequently observed during the case study. Similar observations arose in Italy and Turkey. In Italy, nurses were trained as case managers to manage long-term care processes for chronically ill patients to improve health outcomes. In Turkey, specialised nurses were recruited to counsel patients with type 2 diabetes and support healthy living behaviours to improve their condition. Key skills for an organising professional role are brokering and translating between the hospital and home, and between care and cure.

In short, the main drivers of skill-mix change reside in the technological development of medical treatment and the need for more generic or organising competences in an increasingly complex medical field which offers more treatment on an out-patient basis. While the technical roles focus on the closely specified technical-clinical domain, organising roles concentrate on integrating various aspects of clinical treatment and care and cure activities, which is particularly relevant to care for patients with chronic diseases.

## Discussion

**Fig. 1:**
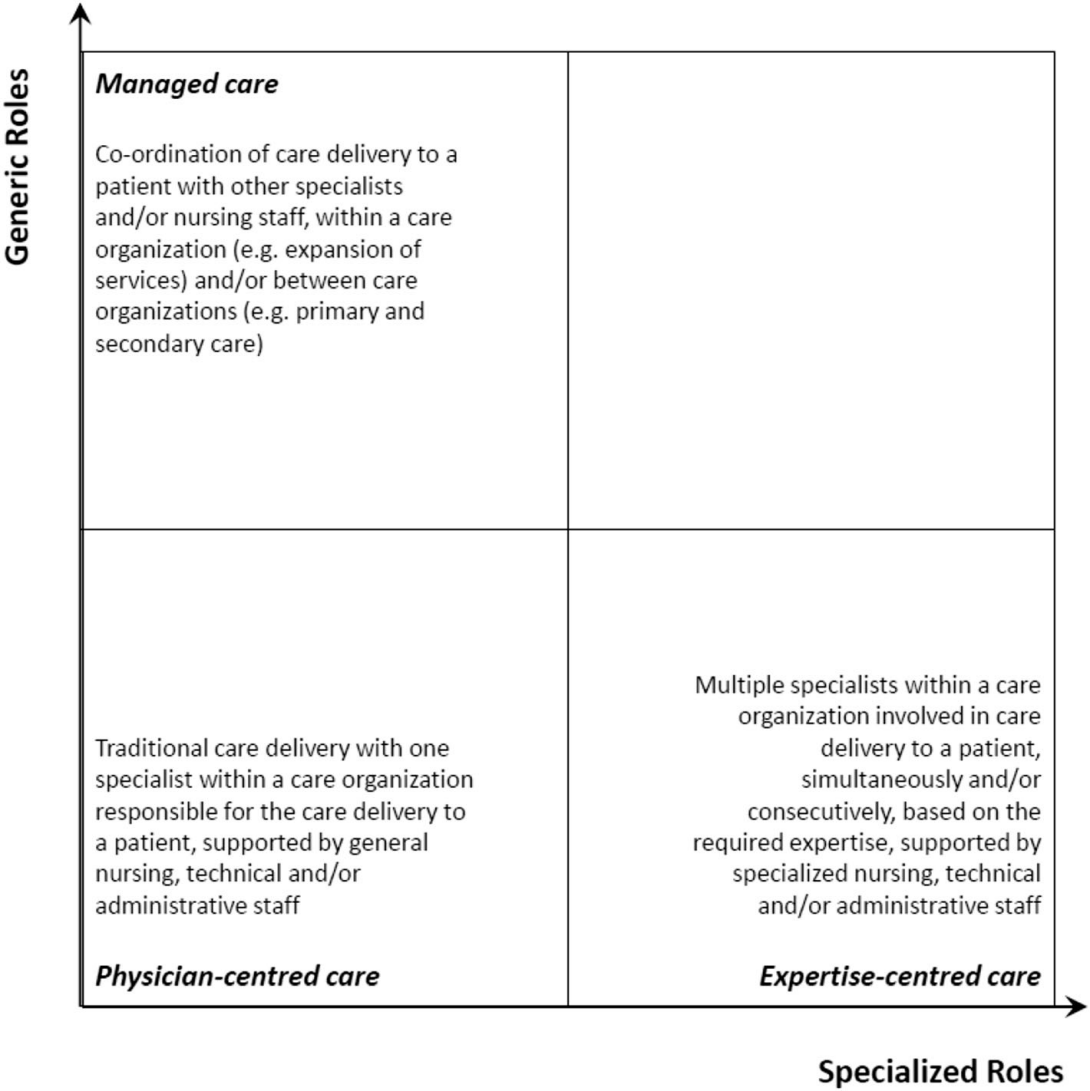
Extended roles in two directions

This paper has revealed a large variety of extended professional roles in three care pathways in eight European countries. In our 16 cases, in total 48 extended professional roles were identified. There are considerable differences in the number and kinds of extended professional roles between and within the three care pathways. Informed by the literature on the sociology of professions and organised professionalism [15, 16, 21], which highlights both professions’ tendency to protect professional jurisdictions and the increasing importance of organizations’ needs in professional role development, we have demonstrated that the extended roles develop in two directions: 1) specialised roles, and 2) generic roles (see Fig. 1). Specialised roles occupy a narrowly defined area of expertise, often concentrating on the medical-technical parts of clinical treatment. Specialised roles are driven by the ever increasing complexity of healthcare services due to medical technological and clinical knowledge development. Generic roles, conversely, have a broader scope and cover a larger part of the care pathway. Generic roles focus on the organisation of care and treatment, often integrating care and cure activities. Generic roles aim to ‘fill up’ the space between the specialised practitioners, guiding the patient through the treatment trajectory. In care delivery, generic roles typically combine and integrate cure and care activities; patients and relatives consult them with questions, and they give advice, considering (drug) treatment as well as dealing with the disease in everyday life.

Extended professional roles can evolve in both specialized and generic directions as they develop particular expertise and clinical routines that contribute to local healthcare quality and efficiency of health service delivery. Moreover, extended roles develop new ways of health service provision that suit healthcare organisations, encouraging and shaping organisation-oriented healthcare delivery.

Role development, this research shows, is a situated endeavour. In contrast to what is expected from initiatives to give nurses extended professional rights and introduce physicians assistants to healthcare systems [36, 37], such formal or legal scope of practice has had less impact to date. Rather, the development of extended roles depends upon the willingness of local physicians (and nurses) to delegate tasks, and the capacities and (perceived) trustworthiness of professionals in these new roles to further develop their work. Personal relationships and trust of a physician in the competences of an individual practitioner play a key role herein [38]. National rules are sometimes unknown, or get overruled by local protocols and guidelines. Moreover, extended roles that lack legal embedding may carry out medical services nonetheless. We have shown how in some cases extended roles (like the paramedic in Poland) carry out clinical activities in a wider range of expertise than formally allowed. In short, extended roles develop, at least partly, independently from the legal context.

Extended roles gain the credibility to perform new and advanced tasks within the teams and organisations they belong to. Local organisational infrastructures drive the introduction and development of new and extended roles rather than professional policies, guidelines and legislation. This also explains why we found fewer differences between countries than expected; although there are many differences – i.e. we identified a wide range of extended roles– extended roles are part of everyday care delivery in all countries.

This ‘locality’ of extended role development has consequences for the employability of these practitioners. As jurisdictions are defined locally, the mobility of practitioners in extended roles is rather limited. In other hospitals other rules and expectations will be applicable for extended roles. Hence, an important question is whether extended role development confines itself to these local practices, or whether the extended roles will find their ‘way up’ to the level of professional associations, educational programs and policy level, leading to formal changes in professional jurisdictions.

There are some limitations of this study that need to be mentioned. First, the findings are based on 16 case studies in eight countries, over three care pathways. Although the sites were selected carefully, we cannot exclude different experiences with new professional roles in the care pathways of other healthcare organisations in the selected countries or in other (non-European) countries. Therefore, this study should be seen as purely indicative of skill-mix developments across Europe, and further study – both qualitative and quantitative – is necessary. Secondly, the case studies were performed by different researchers in each country, which may have led to inconsistencies in data collection or reporting. We have tried to minimise this by organising face-to-face workshops to train all researchers, monthly Skype meetings to exchange experiences with data collection, the use of a standard topic list for interviews and a standard protocol for observations, and by discussing the analysis and findings extensively. Because of this, we are confident about the comparability of the data between countries, consistency in the data analysis, and sensitivity of findings for local differences.

We see at least two relevant avenues for future research. First, it would be interesting to investigate how transferable the findings are to other care pathways and countries. Given the diversity in care pathways and countries selected for this study, similar developments in skill mix and underlying drivers may be observed elsewhere, giving our findings external validity [26]. Second, further research is needed into the effect of diversification of health care teams on the effectiveness and efficiency of care. Although, there is some evidence that skill-mix change may help improve healthcare quality and contain healthcare costs [39], it is largely unknown how different skill-mix arrangements affect outcomes for patients, professionals and the healthcare system, in different care pathways.

## Conclusion

There are considerable differences in the number and kind of extended roles between both countries and care pathways. The main drivers for new roles reside in the technological development of medical treatment and the need for more generic competencies. Extended roles develop in two directions: 1) specialised roles and 2) generic roles.

## Additional files

Additional file 1: Ethnography. (DOCX 40 kb)
Additional file 2: Topic list New professional. (DOCX 32 kb)
Additional file 3: Topic list physicians. (DOCX 29 kb)
Additional file 4: Topic list nurses. (DOCX 29 kb)
Additional file 5: Observation schedule. (DOCX 28 kb)

## Abbreviations

post STEMI: ST-segment elevation myocardial infarction
CCU: Coronary care unit
Cath Lab: Catheterization laboratory
RA: Radiology assistant
CT: Computerized tomography
3D: Three-dimensional
NP: Nurse Practitioner

## Footnotes

1 Health Care Reform: the iMpact on practice, oUtcomes and costs of New roles for health pROfeSsionals (MUNROS project; FP7-HEALTH-2012-INNOVATION-1; http://www.abdn.ac.uk/munros/; @MUNROSproject).
