# Supplementary material for: Reconfiguring health workforce: a case-based comparative study explaining the increasingly diverse professional roles in Europe
Source: BMC Health Serv Res. 2016 Nov 8;16:637. doi: 10.1186/s12913-016-1898-0 (PMC5101691; doi:10.1186/s12913-016-1898-0)
Supplement: Additional file 1: — Ethnography. (DOCX 40 kb) [file 12913_2016_1898_MOESM1_ESM.docx]

**Ethnographic Study in Primary and Secondary Care**

**Introduction**

In the second stage of the research, an ethnographic study is conducted to gain in-depth insight in the daily work of the new and the traditional healthcare professionals in the selected clinical pathways. The research explores:

- The division of work (tasks, responsibilities) and collaboration between health care professionals for the particular clinical pathway (both in, and between primary care and secondary care);
- The competency framework for healthcare professionals involved in the clinical pathway, both in current practice and according to the professional standards and scope of practices as defined by the professional colleges/ other responsible agencies;
- The financial structure and related daily activities to get care remunerated (focus on the particular disease);
- Workforce assessment and workforce planning at the level of healthcare providers;
- The collection and use of outcome data, both in terms of process information and clinical effects.

Case studies are conducted in primary care and in secondary care.

**Research Methods**

The research is an ethnographic study. In addition, a vignette study will be conducted to gain detailed insight in the division of tasks among healthcare professionals in relation to the specific circumstances of the care process. Below, the different research methods are explained. Next, we describe our ethnographic study design.

***Ethnography***

In ethnography, data collection is carried out primarily through fieldwork. Fieldwork can be defined as a form of inquiry that requires a researcher to be immersed personally in the ongoing social activities of some individual or group being studied. Fieldwork allows the researcher to observe and examine all aspects of a certain system, especially those that could not be addressed through laboratory or survey research alone (Wolcott, 1994). It is through observing, interacting with, and participating in their activities that the ethnographer gets an empirically valid understanding of the sociocultural contexts, processes and meaning systems that are of significance to the study participants (Whitehead 2005, Creswell 1998).

Observations can be categorized in descriptive, focused, and selected observations. At the start, usually descriptive observations are conducted to identify general features or phenomena of a social setting. After generating a general impressions of the field, the ethnographic researcher may move on to observe more focused or selected phenomena or situations, exploring the structural characteristics of the domain (Whitehead 2005).

Collected data consists of field notes made during the observations. Field notes are short notes of observed phenomena or situations. Notes are transcribed and elaborated on shortly after the observations, preferably the same day, in order not to lose out on detailed descriptions of observed moments (Pope, 2005).

***Semi-structured interviews***

Besides observations, interviews play a key role in ethnographic research. Interview may be both formal and informal. Informal interviews are, for example, conversations between the researcher and the respondent (the individual being observed). Notes of these conversations are part of the observation transcript (see above).

Formal interviews often have the form of semi-structured interviews, meaning that the interviewer has a written list of questions (‘topic list’) that is used as a guide to have a conversation with a respondent. Topics partly are based on the results of the observations. In semi-structured interviewing, the interviewer elicits answers fully from the perspective of the study participant, and attempts to gain a greater understanding of the context and meaning of those responses through various ways of probing (Whitehead 2005).

Interviewees are defined on base on the ‘purposeful sampling strategy’ (Creswell 1998). Selection is based on the following criteria: work experience, maximum variation, and convenience (Miles and Huberman 1994).

*The interview protocols can be found in Documents D 1-5.*

***Vignette Study***

The ‘case vignette’ study (Busse et al. 2008) aims to explore treatment patterns, and choices in the distribution of work among health professionals. It depicts ‘typical’ patients, including specified age, gender, and relevant co-morbidity. As the vignette method standardizes patients, it can be used for cross-provider and cross-country comparisons, elucidating similarities and differences between national systems of healthcare delivery.

**Study design**

Below we will elaborate on the ethnographic studies in primary and secondary care. The studies are divided in different stages, spelling out the research objectives mentioned in the introduction. For each stage the research approach is described. We start with the description of the research in secondary care, as this research approach is more elaborated. The research in the primary care setting is based on the research approach in secondary care.

**Secondary Care**

**Stage 1**: **Getting Familiar with the Work Environment**

- - Semi-structured interviews with a health care manager (1), a nurse (1), a medical specialist (1), and, if applicable, a new healthcare professional (1), to get familiar with the processes of healthcare provision in the hospital, and workforce related issues.

Selection of respondents: the most experienced healthcare professional is selected for each category. These are the healthcare professionals that are most experienced with the particular clinical pathway in the healthcare institute the case study is conducted.

An interview lasts 30-60 minutes, interviews are transcribed verbatim.

*For the topic list of the semi-structured interview, see Document D.*

**Stage 2: Detecting Healthcare Professional’s Competences regarding the Clinical Pathway**

- On base of available documents, list the care elements for the particular clinical pathway, and list which professional groups perform each element in the studied healthcare institute.
  - **Stage 3**: **Detailed Descriptions of Everyday Healthcare Delivery Processes.**

In this stage, general observations are conducted to give detailed descriptions of the everyday processes of healthcare delivery in the selected clinical pathway. Also, it is investigated whether the division of work among healthcare professionals involved spelled out in Stage 2, maps with the actual process of carrying out clinical activities.

In Stage 3, observations will be conducted from different clinical perspectives: the nurse, the medical specialist, the new healthcare professional, and the patient. Each selected respondent will be shadowed for one day.

The selection is made on basis of experience with the clinical pathway (see Stage 1). In case of the patient, a ‘most convenient sampling approach’ is used. The patient will be selected by the responsible healthcare professional (e.g., physician, nurse or manager). Precondition is that the patient suffers from the selected disease and is admitted to the hospitals because of this disease/ admission is primarily because of the particular disease.

- - 1. Nurse (1 day of observation)
       - Detailed description of daily work
       - Detailed description of collaboration with other health care professionals (colleagues as well as other healthcare workers)
       - Detailed description of activities regarding healthcare finance/reimbursement of healthcare services
    2. Medical specialist (1 day of observation)
- Detailed description of daily work
- Detailed description of collaboration with other health care professionals (colleagues as well as other healthcare workers)
- Detailed description of activities regarding healthcare finance/reimbursement of healthcare services
  - 1. New health care professional (if applicable, 1 day of observation)
       - Detailed description of daily work
       - Detailed description of collaboration with other health care professionals (colleagues as well as other healthcare workers)
       - Detailed description of activities regarding healthcare finance/reimbursement of healthcare services
    2. Patient and (if applicable) relatives (1 day of observation)
       - Detailed description of health care delivery process from patient point of view (experiences, opinions)
       - Detailed description of involvement of relatives in care process
       - Detailed description of which health care professionals take care of the patient during the day (number of patient contacts, time spend)

*During the observations in Stage 3 we examine whether other allied healthcare professionals are involved who can be observed and interviewed in research Phase 6 and 7 (see below).*

*The observation schedules can be found in documents E 1-4*

- - **Stage 5**: **Vignette study**

Interviews on base of developed vignettes to gain insight in workforce related choices and collaboration structures in health care delivery.

- - **Stage 6**: **Focused Observations**

Based on the observations, document analysis and interviews in Stage 1-5, focused observations will be conducted in Stage 6. We focus on:

- Critical points in distribution of tasks, activities and responsibilities;
- Coordination of care among involved healthcare professionals

*The selection of the focused observations will be made on base of the outcomes of Stages 1-5, and will be discussed in the research team, and in collaboration with the leading partner for WP3.*

- - **Stage 7**: **Semi-structured Interviews**

In this final stage, semi-structured interviews are conducted to gain in-depth insight in the division of work, back stage processes of decision making on division of work and related responsibilities, workforce planning, and the reimbursement system. The interviews also are a member check and allow for a discussion of the outcomes of Stages 1-6.

The topic list of the interviews with the healthcare professionals will be based on the observations and subsequent comparative analysis between partner countries. As a consequence, no interview protocol can be provided yet.

The interview protocol for the human resource manager and financial manager (see below) can be found in Document D.

Interviews are conducted with:

- - 1. Medical specialist (1, another physician than in Stage 1)
    2. Nurse (1, another nurse that in Stage 1)
    3. New healthcare professional (if applicable, another new healthcare professional than in Stage 1)
    4. Healthcare manager and/or executive (1-2, not the same respondents as in Stage 1)
    5. Human Resource Manager (1)
    6. Financial manager or other employee involved in finance health care services (1-3, depending on how many financial workers in different positions are involved)

*The interview protocols can be found in Documents D 6-7*

**Primary care**

The research in the primary care setting basically follows the format of the research methods used in secondary care. However, from the outcomes of WP1 and 2, as well as other research projects, it is expected that the research in primary care will be less systematically as we do not expect to come across patients with the selected disease every day, unless there is a specialized healthcare professional working in GP practice (e.g. a diabetes nurse).

- - **Stage 1**: Interview with 1-2 general practitioners (depending on the size of the GP practice) and, if applicable, a nurse and/ or doctor’s assistant working in GP practice. General aim is to get familiar with the processes of healthcare provision in GP practice, and workforce related issues. Special attention is paid to the collaboration with secondary care for the selected clinical pathway.

**Stage 2**: **Detecting Healthcare Professional’s Competences regarding the Clinical Pathway**

- On base of available documents, list the care elements for the particular clinical pathway, and list which professional groups perform each element in the studied GP practice.

**Stage 3**: Gain in-depth insight in, and provide detailed descriptions of everyday health care delivery processes in GP practice. Also, it is investigated whether the division of work among healthcare professionals spelled out in Stage 2, maps with the actual process of carrying out clinical activities.

Observations are conducted from different clinical perspectives:

- - 1. General practitioner (1 day of observation)
       - Detailed description of daily work
       - Detailed description of collaboration with other health care professionals (colleagues as well as other healthcare workers in GP practice)
       - Detailed description of activities related to healthcare finance/reimbursement of provided services
    2. Nurse working in GP practice (1 day of observation)
       - Detailed description of daily work
       - Detailed description of collaboration with other health care professionals, in particular the GP
       - Detailed description of activities regarding healthcare finance/reimbursement of provided services
    3. If applicable: specialized nurse/ other allied healthcare professional involved in selected clinical pathway (1 day of observation)
       - Detailed description of daily work
       - Detailed description of collaboration with other health care professionals, in particular the GP
       - Detailed description of activities regarding healthcare finance/reimbursement of provided services
    4. Doctor’s assistant (if applicable, and if conducting clinical tasks, 1 day of observation)
       - Detailed description of daily work
       - Detailed description of collaboration with other health care professionals, in particular the GP
       - Detailed description of activities regarding healthcare finance/reimbursement of provided services
  - **Stage 4:** **Vignette Study**

Interviews on base of developed vignettes to gain insight in workforce related choices and collaboration structures in health care delivery.

- - **Stage 5**: **Semi-structured interviews**

Semi-structured interviews are conducted to gain in-depth insight in the division of work, and related responsibilities, workforce planning, and the financial system. Besides, the interviews are a member check, and allow for a discussion of the outcomes of Stages 1-4. Interviews are conducted with:

- - 1. General practitioner (1)
    2. Nurse (1, if applicable)
    3. Specialized nurse/other allied healthcare professional (if applicable)
    4. Healthcare manager or executive (if applicable)
    5. HRM manager (if applicable)
    6. Financial manager or other employee involved in finance health care services (if applicable)

*Selection of GP practice:* A GP practice is selected in which a specialized nurse or another allied health care professional is involved in the treatment of patients with the particular disease. If such GP practice is not available, a convenient general GP practice is selected.

**References**

Busse, R., Schreyögg, J., and Smith, P.C. (2008). Editorial. Variablity in healthcare treatments costs amongst nine EU countries- Results from the *Health*Basket project. *Health Economics*, 17:S1-S8.

Creswell, J.W. (1998). Qualitative inquiry and research

Miles, M.C., and Huberman, A.M. (1994). *Qualitative data analysis: A sourcebook of new methods* (2^nd^ ed.). Thousand Oaks, CA: Sage.

Pope, C. Conducting ethnography in medical settings. *Medical Education*, 39: 1180-87.

Whitehead, T.L. (2005). *Basic classical ethnographic research methods: Secondary data analysis, fieldwork, observation/participant observation, and informal and semi-structured interviewing*. CEHC: Cultural Ecology of Health and Change.

Wolcott, H.F. (1994). *Transforming qualitative data: Description, analysis, and interpretation*. Thousand Oaks, CA: Sage.
