# Supplementary material for: Reconfiguring health workforce: a case-based comparative study explaining the increasingly diverse professional roles in Europe
Source: BMC Health Serv Res. 2016 Nov 8;16:637. doi: 10.1186/s12913-016-1898-0 (PMC5101691; doi:10.1186/s12913-016-1898-0)
Supplement: Additional file 2: — Topic list New professional. (DOCX 32 kb) [file 12913_2016_1898_MOESM2_ESM.docx]

**Interview Protocol New Professional**

**Interview Protocol New professional**

Project: Munros

Define new professional (profession):

Time of interview:

Date:

Place:

Interviewer:

Interviewee:

**Questions**:

1. What are the tasks and responsibilities of the new professional in the clinical pathway? Both on paper and in current practice?
2. How is the care provided by the new professional in the clinical pathway adjusted to other healthcare workers (nurses, physicians, other allied health professionals) within the particular healthcare institute?
3. Have any changes been made to the clinical pathway in terms of the distribution of tasks and responsibilities between professional groups in recent years?
4. Are the competences the new professional has been trained for, fully made use of in the delivery of care?
5. Does integration of care for this pathway exist? If yes, to what extent and in what ways?
6. How does the integration of care affect the tasks and responsibilities of the new professional?
