# Supplementary material for: Reconfiguring health workforce: a case-based comparative study explaining the increasingly diverse professional roles in Europe
Source: BMC Health Serv Res. 2016 Nov 8;16:637. doi: 10.1186/s12913-016-1898-0 (PMC5101691; doi:10.1186/s12913-016-1898-0)
Supplement: Additional file 4: — Topic list nurses. (DOCX 29 kb) [file 12913_2016_1898_MOESM4_ESM.docx]

**Interview Protocol Nurse, Ethnographic Research**

**Interview Protocol Nurses Stage 1**

Project: Munros

Time of interview:

Date:

Place:

Interviewer:

Interviewee:

**Questions**:

1. What are the tasks and responsibilities of nurses in the clinical pathway? Both on paper and in current practice?
2. How are these tasks distributed among the group of nurses?
3. How is the care provided in the clinical pathway adjusted to other healthcare workers (physicians, allied health professionals) within the particular healthcare institute?
4. Have any changes been made to the clinical pathway in terms of the distribution of tasks and responsibilities between professional groups in recent years?
5. Does integration of care for this pathway exist? If yes, to what extent and in what ways?
6. How does the integration of care affect the tasks and responsibilities of nurses?
