# Supplementary material for: Reconfiguring health workforce: a case-based comparative study explaining the increasingly diverse professional roles in Europe
Source: BMC Health Serv Res. 2016 Nov 8;16:637. doi: 10.1186/s12913-016-1898-0 (PMC5101691; doi:10.1186/s12913-016-1898-0)
Supplement: Additional file 5: — Observation schedule. (DOCX 28 kb) [file 12913_2016_1898_MOESM5_ESM.docx]

**Document E**

**Observation schedule Ethnographic Research**

Date of Observation:

Place:

Researcher:

Professional being observed:

Duration of observation: ….hours

| **Involvement in care process** | **Activities** | **Location/ describe situation** | **Collaboration with other health professionals/ care takers (e.g., consultation, delegation of tasks, adjustment, coordination)** | **Additional observations** |
| --- | --- | --- | --- | --- |
| Diagnostic phase   - diagnosis and assessment - providing information & support - otherwise… |  |  |  |  |
| Treatment   - medical treatment - providing medication - wound care - otherwise… |  |  |  |  |
| Managing complications   - wound infections - otherwise…. |  |  |  |  |
| Supportive care   - patient - relatives/ informal care givers |  |  |  |  |
| Administrative tasks  - |  |  |  |  |
| Financial administration |  |  |  |  |
| Professional development |  |  |  |  |
| Other….[please define!] |  |  |  |  |
